# Comparison of prioritisation algorithms for the selection of patients for medication reviews in the emergency department: a cross-sectional study

**DOI:** 10.1007/s11096-023-01582-0

**Published:** 2023-04-20

**Authors:** Signe Gejr Korup, Anna Birna Almarsdóttir, Line Magnussen

**Affiliations:** 1 Social and Clinical Pharmacy Research Group, Department of Pharmacy, Faculty of Health and Medical Sciences, University of Copenhagen, Universitetsparken 2, 2100 Copenhagen, Denmark; 2 Capital Region Hospital Pharmacy, Nordsjællands Hospital, Dyrehavevej 29, 3400 Hillerød, Denmark

**Keywords:** Assessment of risk tool, Drug-related problems, Medication review, Medicine risk score, Risk assessment

## Abstract

**Background:**

Risk prioritisation algorithms provide patients with a risk category that guides pharmacists to choose those needing medication reviews (MRs) the most. For this study the Medicine Risk Score (MERIS) and a modified Assessment of Risk Tool (ART) were used.

**Aim:**

To examine how the selection of patients by the clinical pharmacists in an emergency department for MRs compared with the categorisation provided by MERIS and a modified version of ART (mART). Furthermore, examine the agreement between MERIS and mART.

**Method:**

A cross-sectional study was conducted using data on all admitted patients during a two-month period. Data were entered into the prioritisation algorithms and independently ranked by the six pharmacists who were observed as they selected patients for MR. Risk scores and categorisations were compared between the algorithms and the pharmacists’ ranking using t-test, Z-test, Chi square, Kruskal Wallis H-test, or Kappa statistics.

**Results:**

The study included 1133 patients. Significant differences were found between the pharmacists and the algorithms. The sensitivity and specificity of MERIS were 37.8% and 73.6%, for mART, 33.0% and 75.9%. Kappa was 0.58, showing moderate agreement. No significant differences were observed between the individual pharmacists’ selection, but differences were significant between how pharmacists ranked the importance of the provided MRs.

**Conclusion:**

Pharmacists disagreed with the risk categorisation provided by MERIS and mART. However, MERIS and mART had similar sensitivity, specificity, and moderate agreement. Further research should focus on how clinical algorithms affect the selection of patients and on the importance of the MRs carried out by pharmacists.

**Supplementary Information:**

The online version contains supplementary material available at 10.1007/s11096-023-01582-0.

## Impact statements

- Non-clinical ground rules disagree with risk prioritisation algorithms that are based on clinically important risk factors.
- This comparison can be used to provide pharmacists with a better foundation on which to decide between risk prioritisation algorithms in a clinical setting.
- Medicine Risk Score and a modified version of Assessment of Risk Tool had a moderate agreement in their risk categorisation.

## Introduction

Medication reviews (MRs) aim to improve the quality, safety, and appropriate use of medications by finding and solving drug-related problems (DRP) [1–4]. Important risk factors for DRPs include polypharmacy, female gender, high risk medications, renally eliminated drugs and being elderly (most often 65 years of age and older) [2, 3]. Some of these risk factors are also used as criteria for patient prioritisation for MRs [5, 6].

At the joint emergency department (JED) at Nordsjællands Hospital in Denmark, pharmacists provide medication reviews (MRs) to hospitalised patients. Risk prioritisation algorithms could help find patients for whom the pharmacist's resources are best utilised [7, 8]. Several risk prioritisation algorithms have been developed, such as the Brighton Adverse Drug Reactions Risk model and the Medicines Optimisation Assessment Tool. These risk prioritisation algorithms include different items used to calculate the risk of DRP or measure other similar outcomes [9–20].

The JED at Nordsjællands Hospital has a high patient flow and only one pharmacist providing MRs on any given day, so it was essential to select the patients that would benefit the most from MRs [7, 21]. Currently, pharmacists at JED do not use a prioritisation algorithm but use non-clinical ground rules developed by JED. The non-clinical ground rules prioritise patients with the longest stay and who have not had their medications transferred to the electronic health record (EHR) [22]. Therefore, it was important to examine the current non-clinical ground rules and compare current practice with the risk categorisation of the prioritisation algorithms.

This study used the algorithm Medicine Risk Score (MERIS) developed in Denmark using literature search and the Delphi method and a modified version of Assessment of Risk Tool (ART) developed in New Zealand using a group consensus method. MERIS consists of three items: *reduced renal function, number of drugs* and *number of drugs with different categories of risk of harm and risk of interaction*. ART consists of 38 items divided into five categories: *patient profile, patient encounter, clinical profile—patients with chronic diseases, high-risk medications* and *laboratory values.* Both algorithms provide risk categories by adding points from the fulfilled items [23–26].

### Aim

To examine how the selection of patients by the clinical pharmacists in an emergency department for MRs compared with the categorisation provided by MERIS and a modified version of ART (mART). Furthermore, examine the agreement between MERIS and mART.

### Ethics approval

No ethical approval was required according to Danish law [27], however the pharmacists were anonymous and all material was stored confidentially. Data collection and processing were in compliance with the European General Data Protection.

## Method

### Study design

A cross-sectional study was conducted at the JED at Nordsjællands Hospital in Denmark. Patient data relevant for the algorithms were collected while observing the pharmacists working at the JED, who provided MRs daily from 8 a.m. to 5 p.m. All admitted patients 18 years of age and older were included the study, as well as patients who stayed overnight and patients admitted until approximately 4:45 p.m. Afterwards, patient data were entered into the risk prioritisation algorithms MERIS and the modified ART (mART). When ART was developed, it had 38 items [25]. However, some of these items were specific to the New Zealand health care setting or not suitable for a fully automatic algorithm, or relevant in the JED. Therefore, 12 items were removed to make the mART used in this study. Examples of items removed were: being from specific New Zealand native populations, poor English proficiency, history of psychiatric disease, and having specific laboratory values taken over several days. See electronic supplementary material 1 for the mART items.

### Data collection

The first author (SGK), a student in her last semester prior to graduating as a pharmacist, followed six pharmacists working at JED. Each pharmacist was observed for three days from March 9th to May 6th, 2022, on weekdays and weekends. The pharmacists ranked their MRs as ‘not important’, ‘important’ or ‘very important’, based on their clinical insight. The pharmacists did not change their typical workday during the data collection, meaning that they continued to attend meetings and precept students. These days, they did not manage to prioritize and complete as many MRs as they might have otherwise. The overall study included both the patients who were actively deselected for MRs and those admitted patients whom the pharmacists did not have time to consider.

The algorithms Medicine Risk Score (MERIS) and a modified version of Assessment of Risk Tool (mART) were used. Besides the relevant information for the different items in MERIS and mART, information about the patients’ age and gender were collected. The patients’ medication lists were extracted manually from the EHR and entered into an Excel spreadsheet. All medication formulations were counted (i.e. tablets, cremes, eyedrops). Duplicate medications and combination medications were counted once per active ingredient. The medication lists were taken from the EHR and not from patient interviews. Unless otherwise stated it was assumed that every drug on the list was used by the patient.

### Data analysis

A frequency analysis of the total mART risk scores made the cut-off values for the risk categories of the mART. As observed in the original ART development the 10%, 15%, and 75% cut-offs were used [25]. The mART cut-off values were based on a frequency analysis of the total ART risk scores. Patients with a risk score above 36 were defined as high-risk, 25–36 as medium-risk, and 24 or under were low-risk. In the original version of ART the cut-off values were: above 22 were defined as high-risk, 11–22 as medium-risk, and 10 or under were low-risk [25].

The mean ages of patients in the high versus those in the low-risk categories of the algorithms were compared as were mean risk scores of patients who had either been selected or deselected by the pharmacists. Proportions of females were compared in the algorithms' high versus low-risk categories. Finally, the pharmacists’ ratings of the importance of the MRs were compared to the risk categorisations provided by the algorithms.

The sensitivity and specificity of the MERIS and mART were calculated using the pharmacists’ selection and deselection as the gold standard. For mART the medium and high-risk categories were merged. A true positive and a true negative were when the algorithms and the pharmacists agreed and a false positive and a false negative when they disagreed. The sensitivity, specificity and Kappa were calculated twice, respectively including and excluding patients with missing laboratory values.

The pharmacists’ numbers of patients selected and deselected for MRs were compared among them and their risk categories against MERIS and mART. In addition, the ratings of importance were compared among the individual pharmacists.

T-tests were used for comparing means, Z-tests for proportions. The Kappa Statistics test assessed the agreement between the algorithms’ risk categorisation. Finally, Kruskal–Wallis H, Chi square and Monte Carlo significance tests were used for nominal variables. All statistical analyses were done in SPSS version 28.0.0.0.

## Results

In total, 1133 patients were included in the analysis of MERIS and mART, where pharmacists actively selected or deselected from 450 patients. Approximately 70% of the patients were categorised as low-risk on both the MERIS and mART tools (Table 1).

**Table 1:** Distributions of patients included in the study in each risk category with the original ART, the modified ART, and MERIS (n = 1133)

	Distribution of patients, n (%)		
	MERIS	Modified ART	Original ART
High-risk	336 (29.7)	118 (10.4)	354 (31.2)
Medium-risk	N/A	193 (17.0)	296 (26.1)
Low-risk	797 (70.3)	822 (72.6)	483 (42.6)

Statistically significant differences were observed in the mean age between the risk categories for MERIS and mART, where patients classified as high-risk had a higher mean age. Similarly, patients selected by pharmacists had a higher mean age than those deselected for MRs. No statistically significant gender differences were observed with this categorisation (Table 2).

**Table 2:** Characteristics of patients in the study according to risk categories in MERIS and the modified ART and according to whether they were selected or deselected for medication review by pharmacists

Age, mean (SD)			Test statistic (*p* value)
MERIS	High-risk	74.7 (13.8)	t = − 10.29 (< 0.001)
	Low-risk	62.0 (20.9)	
	Total, n	1133	
Modified ART	High-risk	78.2 (11.7)	t = − 8.69 (< 0.001)
	Medium-risk	76.7 (12.8)	
	Low-risk	61.4 (20.5)	
	Total, n	1133	
Pharmacists	Selected	69.8 (17.9)	t = − 3.90 (< 0.001)
	Deselected	62.6 (21.5)	
	Total, n	450	

Gender females, n (%)			Test statistic (*p* value)
MERIS	High-risk	180 (53.6)	z = 0.92 (0.358)
	Low-risk	403 (50.6)	
	Total, n	1133	
Modified ART	High-risk	53 (44.9)	z = 1.35 (0.177)
	Medium-risk	106 (54.9)	
	Low-risk	424 (51.6)	
	Total, n	1133	
Pharmacists	Selected	130 (56.5)	z = 1.96 (0.050)
	Deselected	104 (47.3)	
	Total, n	450	

No statistically significant differences were found between the risk scores of selected and deselected patients for MRs in the high-risk category of MERIS, indicating that the risk scores were similar. This also applied to the high-risk and medium-risk categories of mART. However, for the low-risk categories of MERIS and mART, statistically significant differences were seen between patients who were selected and deselected by pharmacists, where the highest risk score was observed in the selected patients (Table 3).

**Table 3:** Risk scores of the patients who were selected or deselected by pharmacists for medication review in each of the risk categories of MERIS and the modified ART (n = 450)

		MERIS		
		Patients, n (%)	Risk score, mean (SD)	Test statistic (*p* value)
High-risk	Selected	84 (18.7)	18.6 (3.9)	t = 1.23 (0.221)
	Deselected	58 (12.9)	19.4 (3.9)	
Low-risk	Selected	146 (32.4)	6.5 (3.9)	t = − 3.99 (< 0.001)
	Deselected	162 (36.0)	4.6 (4.4)	

		Modified ART		
		Patients, n (%)	Risk score, mean (SD)	Test statistic (*p* value)
High-risk	Selected	29 (6.4)	46.0 (7.4)	t = − 1.40 (0.168)
	Deselected	18 (4.0)	43.1 (5.4)	
Medium-risk	Selected	46 (10.2)	30.3 (3.4)	t = 1.29 (0.200)
	Deselected	35 (7.8)	31.3 (3.3)	
Low-risk	Selected	155 (34.4)	11.0 (7.9)	t = − 3.09 (0.002)
	Deselected	167 (37.1)	8.3 (7.7)	

A statistically significant difference was observed between the pharmacists’ rating of importance of providing MR to the patient and the categorization of patients by both algorithms. However, for both algorithms there was a larger number of ‘not important’ ratings by the pharmacists in the low-risk categories, indicating that there might be some agreement between the pharmacists and low-risk categorisation by the algorithms (Table 4).

**Table 4:** The pharmacists’ rating of the importance of patients receiving a medication review compared to the different risk categories of MERIS and the modified ART (n = 230)

*Pharmacists*			
	MERIS high-risk, n (%)	MERIS low-risk, n (%)	Test statistic (*p* value)
Very important	21 (9.1)	15 (6.5)	Χ^2^ = 11.40 (0.003)
Important	34 (14.8)	54 (23.5)	
Not important	29 (12.6)	77 (33.5)	

*Pharmacists*				
	Modified ART high-risk, n (%)	Modified ART medium-risk, n (%)	Modified ART low-risk, n (%)	Test statistic (*p* value)
Very important	12 (5.2)	10 (4.3)	14 (6.1)	Χ^2^ = 27.49 (< 0.001)
Important	11 (4.8)	22 (9.6)	55 (23.9)	
Not important	6 (2.6)	15 (6.5)	85 (37.0)	

The sensitivity and specificity for MERIS were 37.8% and 73.6%, respectively when the patients with missing laboratory values were included. When these patients were removed the values were 37.9% and 72.0%. The sensitivity and specificity for mART were 33.0% and 75.9% respectively, and 33.3% and 74.0% when removing patients with missing laboratory values. The agreement of risk categorisation between MERIS and mART (grouping the high-risk and medium-risk groups of mART) was statistically significant (Kappa = 0.58), indicating a moderate agreement [28]. The same applied to patients when excluding patients with missing laboratory values (Table 5).

**Table 5:** Distribution of patients across the different risk categories comparing MERIS and the modified ART, both when patients with missing laboratory values were included and excluded

*Patients with missing laboratory values included, n* = *1133*				
		MERIS, n (%)		Test statistic (*p* value)
		High-risk	Low-risk	
Modified ART	High + medium-risk	227 (2.0)	85 (7.5)	Κ = 0.58 (< 0.001)
	Low-risk	110 (9.7)	711 (62.8)	

*Patients with missing laboratory values excluded, n* = *1015*				
		MERIS, n (%)		Test statistic (*p* value)
		High-risk	Low-risk	
Modified ART	High + medium-risk	212 (20.9)	77 (7.6)	K = 0.58 (< 0.001)
	Low-risk	100 (9.9)	626 (61.7)	

High and medium-risk groups of the modified ART were merged

When looking at the items of mART, three were not fulfilled by any of the patients. These were the following: *a positive Clostridium difficile toxin culture*, *a therapeutic drug monitoring test,* and *an activated partial thromboplastin time of over 100 s*. Generally, the items relating to laboratory were fulfilled by a low proportion of patients. The *estimated glomerular filtration rate (eGFR)* < *30 ml/min* and *P-glucose* > *11 mmol/L* were the items most frequently used (5.7% and 3.6% of all patients). The most frequently fulfilled mART items were: *having more than 8 drugs* and *being over 75 years old* (approximately 40% of the patients). In MERIS every item was fulfilled by at least one patient. The most frequently fulfilled items were *drugs with low and medium risk of interaction* and *drugs with medium risk of harm* (96.9% and 82.1%).

No statistically significant difference was observed between the individual pharmacists’ number of selected and deselected patients. However, Pharmacist 3 stood out as selecting the most patients for MRs and deselecting the fewest patients. A statistically significant difference between the individual pharmacists’ rating of importance of the provided MRs was observed. Pharmacist 1 had the largest proportion of ‘very important’ MRs, whereas Pharmacist 3 had the largest proportion of ‘not important’ MRs. No statistically significant difference between the individual pharmacist’s number of MRs in each risk category of the two algorithms. For most of the pharmacists there was a larger proportion of MRs in the low-risk category of both algorithms (Table 6).

**Table 6:** Characteristics of the individual pharmacists in the study according to selection or deselection of patients for medication reviews, rating of importance, and the risk categories of MERIS and ART (n = 230)

*Patients, n (%)*				
Pharmacist experience	Selected for medication review	Deselected	Patients, n	Test statistic (*p* value)
1, < 2 years	26 (42.6)	35 (57.4)	61	H = 2.99 (0.084)
2, < 2 years	43 (43.9)	55 (56.1)	98	
3, 3—4 years	51 (78.5)	14 (21.5)	65	
4, 3—4 years	32 (36.4)	56 (63.6)	88	
5, > 4 years	32 (52.5)	29 (47.5)	61	
6, > 4 years	46 (59.7)	31 (40.3)	77	

*Rating of importance, n (%)*				
	Very important	Important	Not important	Test statistic (*p* value)
1, < 2 years	10 (38.5)	7 (26.9)	9 (34.6)	Χ^2^ = 23.93 (0.004)
2, < 2 years	5 (11.6)	18 (41.9)	20 (46.5)	
3, 3–4 years	5 (9.8)	14 (27.5)	32 (62.7)	
4, 3–4 years	6 (18.8)	9 (28.1)	17 (53.1)	
5, > 4 years	5 (15.6)	16 (50.0)	11 (34.4)	
6, > 4 years	5 (10.9)	24 (52.2)	17 (37.0)	

*MERIS, n (%)*			
	High-risk	Low-risk	Test statistic (*p* value)
1, < 2 years	12 (46.2)	14 (53.8)	H = 9.56 (0.089)
2, < 2 years	10 (23.3)	33 (76.7)	
3, 3–4 years	18 (35.3)	33 (64.7)	
4, 3–4 years	11 (34.4)	21 (65.6)	
5, > 4 years	18 (56.3)	14 (43.8)	
6, > 4 years	18 (39.1)	28 (60.9)	

*mART, n (%)*				
	High-risk	Medium-risk	Low-risk	Test statistic (*p* value)
1, < 2 years	3 (11.5)	9 (34.6)	14 (53.8)	H = 7.68 (0.175)
2, < 2 years	5 (11.6)	7 (16.3)	31 (72.1)	
3, 3– years	4 (7.8)	8 (15.7)	39 (76.5)	
4, 3–4 years	7 (21.9)	6 (18.8)	19 (59.4)	
5, > 4 years	6 (18.8)	8 (25.0)	18 (56.3)	
6, > 4 years	4 (8.7)	9 (19.6)	33 (71.7)	

## Discussion

### Statement of key findings and interpretation

The cut-off values of mART corresponded to the distribution of patients in MERIS. Since mART had fewer items than the original ART and the maximum score was lower, naturally the risk category cut-off values changed. The mART cut-off values were higher than those of the original algorithm, which could be due to this patient population having higher risk scores than the population used in the development of original ART.

A higher mean age in the high-risk categories is not surprising as high age is an item in mART. Higher age is associated with using more medications, which could explain why those in the high-risk category of MERIS also had a higher mean age, as MERIS includes *number of drugs* as an item, but not age [29]. The same difference was observed in the pharmacists’ selection, indicating that they may believe that older patients are more likely to have a DRP. No gender differences were observed in the risk categories, despite other studies showing that more women experienced polypharmacy [29–31].

Patients selected or deselected by pharmacists were at a similar risk of a DRP according to MERIS and mART. This indicates that the pharmacists disagreed with the risk categorisation provided by the algorithms. The non-clinical ground rules could also have influenced the selection and deselection, as the rules did not consider clinical risk factors [5]. It could also be a coincidence that the deselected patient had high risk scores since they were deselected for various reasons. However, in the low-risk categories of MERIS and ART significant differences were observed between the selected and deselected patients, where the selected had a higher risk score. This indicates that the pharmacists selected patients at higher risk within the low-risk category. The study also showed differences between the pharmacists’ rating of importance and the risk categorisation provided by the algorithms, indicating that they disagreed with the risk categorisation.

The sensitivity and specificity of MERIS and mART were similar, indicating an agreement in the risk categorisation by the algorithms. Previous studies found that the sensitivity and specificity of MERIS were 64 to 81% and 52 to 75% [23, 32]. The specificity of MERIS for this study was similar. However, the sensitivity was lower, which could be due to using the pharmacists as the gold standard as their non-clinical ground rules did not necessarily take the risk of DRP into account. No sensitivity and specificity values have been reported earlier for mART. However, the same issue of the pharmacists’ non-clinical ground rules could also influence the relatively low sensitivity found in this study. A moderate agreement of risk categorisation by MERIS and mART was found. With the distribution of patients across the risk categories, it seems probable that the algorithms would agree since approximately 30% were at high-risk, and 70% were at low-risk.

The laboratory items of the mART were the items most often missing from the EHR. It could be due to the study taking place at the JED, where the patients did not stay for long and few had blood work done. Including many laboratory items is probably not applicable in this setting since mART was not explicitly developed for emergency departments [25]. The most fulfilled items of mART match those from its validation study [26]. All the items of MERIS were fulfilled and easy to collect from the EHR compared to mART.

Differences were found between the individual pharmacists in the number of patients they each selected or deselected for MRs. Research indicates an association between clinical experience and how pharmacists prioritise patients, where the more experienced often take the reason for admission into account [22]. Clinical experience did not seem to be a factor in how pharmacists prioritized patients in this study, although pharmacists with the least clinical experience rated the largest proportion of patients as ‘very important’, indicating that they may generally feel more responsibility for providing MRs. All pharmacists rated a relatively large proportion of patients as ‘not important’, which again could be due to their non-clinical ground rules. Differences between pharmacists could be because they made decisions at different speeds or some did not necessarily follow the non-clinical ground rules, or due to alternating workdays with administrative or education tasks.

No difference was observed between the individual pharmacists’ number of MRs in the different risk categories of the algorithms. However, all the pharmacists had a larger number of MRs for patients in the low-risk categories, which could be due to their adhering to the non-clinical ground rules. This could also be due to the algorithms' categorisation of approximately 70% of patients in the low-risk category, which could explain why a higher proportion of low-risk patients were selected by pharmacists.

### Strengths and limitations

One of the study’s strengths was that the data were collected on three alternate days for each pharmacist to get a larger data set with fewer day-to-day variations. To our knowledge a comparison of MERIS and ART has not been done before on the same patient population.

A limitation of the study was that the medication lists were based on the EHR, which was not always correct or updated. A recent Danish study has shown that 81% of the patients included had at least one discrepancy between EHR and what the patients reported taking at home [33]. If this applied to the patients in this study, it could bias the risk categorisation. This could have been avoided by basing the medication lists on patient interviews. Also, the pharmacists did not rate the same patients, so it was not possible to conclude strongly about their similarities or differences. An independent expert could have rated the patients for a more objective comparison. However, since the pharmacists were supposed to work according to the same non-clinical ground rules and provide a similar performance, it seemed reasonable to put them in the same group to get a more realistic picture. Conducting this study only in one ED is a limitation and needs to be considered if these results are transferred.

A further limitation was a potential observer effect, as the pharmacists knew that the observer was noting the number of patients selected and deselected for MRs. This could have influenced how the pharmacists worked by making them work faster and selecting a higher number of patients than they normally would. The observation could also have influenced which patients the pharmacists selected, although it was not possible to know in which way this would affect the results.

### Further research

Future research should have the pharmacists use one of these risk algorithms to examine how it affects their prioritisation and the importance of the MRs they then provide. It could be interesting to further characterize the patients where the algorithms disagreed in the risk categorisation and delve into the disagreements between the pharmacists’ categorizations and risk algorithms. Further work should also examine if there could be an association between the risk categorisation by the algorithms and the reason for admission.

## Conclusion

The study found that the pharmacists disagreed with the risk categorisation provided by MERIS and mART. This could be due to the pharmacists' non-clinical ground rules, which did not consider clinical risk factors when selecting patients for MRs. However, MERIS and mART were found to have similar sensitivity, specificity, and moderate agreement in their risk categorisation. Further research should focus on how clinical algorithms affect the selection of patients and the importance of the MRs carried out by pharmacists.

## Supplementary Information

Below is the link to the electronic supplementary material.

Supplementary file1 (PDF 121 KB)

## References

[1] Blenkinsopp A, Bond C, Raynor DK (2012). Medication reviews. Br J Clin Pharmacol.

[2] Krähenbühl-Melcher A, Schlienger R, Lampert M (2007). Drug-related problems in hospitals a review of the recent literature. Drug Saf.

[3] van den Bemt PM, Egberts TC, de Jong-Van Den Berg LT (2000). Drug-related problems in hospitalised patients. Drug Saf.

[4] Hellebek A, Marinakis C. Patientsikkerhed og lægemidler. Available from: https://pro.medicin.dk/specielleemner/emner/550. Accessed 01 Jun 2022.

[5] Botelho SF, Pantuzza LLN, Marinho CP (2022). Consensus on the criteria for patient prioritization in hospital clinical pharmacy services: a Delphi study. Int J Clin Pharm.

[6] Linkens AEMJH, Janssen MJM, van Nie N (2022). Additional value of a triggerlist as selection criterion in identifying patients at high risk of medication-related hospital admission: a retrospective cohort study. Int J Clin Pharm.

[7] Fernandes O, Shojania KG (2012). Medication reconciliation in the hospital: what, why, where, when, who and how. Healthc Q.

[8] Lewis P (2017). Right patient, right time, right pharmacist: the time for clinical prioritisation tools?. Eur J Hosp Pharm.

[9] Sakuma M, Bates DW, Morimoto T (2012). Clinical prediction rule to identify high-risk inpatients for adverse drug events: the JADE Study. Pharmacoepidemiol Drug Saf.

[10] Crutzen S, Schuling J, Hugtenburg JG (2019). Development and piloting of an algorithm to select older patients for different types of medication review. Front Pharmacol.

[11] Kiguba R, Karamagi C, Bird SM (2017). Incidence, risk factors and risk prediction of hospital-acquired suspected adverse drug reactions: a prospective cohort of Ugandan inpatients. BMJ Open.

[12] Alshakrah MA, Steinke DT, Tully MP (2021). Development of the adult complexity tool for pharmaceutical care (ACTPC) in hospital: a modified Delphi study. Res Social Adm Pharm.

[13] Hohl CM, Partovi N, Ghement I (2017). Impact of early in-hospital medication review by clinical pharmacists on health services utilization. PLoS ONE.

[14] Hohl CM, Yu E, Hunte GS (2012). Clinical decision rules to improve the detection of adverse drug events in emergency department patients. Acad Emerg Med.

[15] de Winter S, Vanbrabant P, Laeremans P (2017). Developing a decision rule to optimise clinical pharmacist resources for medication reconciliation in the emergency department. Emerg Med J.

[16] Pippins JR, Gandhi TK, Hamann C (2008). Classifying and predicting errors of inpatient medication reconciliation. J Gen Intern Med.

[17] Urbina O, Ferrández O, Grau S (2014). Design of a score to identify hospitalized patients at risk of drug-related problems. Pharmacoepidemiol Drug Saf.

[18] Geeson C, Wei L, Franklin BD (2019). Development and performance evaluation of the Medicines Optimisation Assessment Tool (MOAT): a prognostic model to target hospital pharmacists’ input to prevent medication-related problems. BMJ Qual Saf.

[19] Geeson C, Wei L, Franklin BD (2017). Medicines Optimisation Assessment Tool (MOAT): a prognostic model to target hospital pharmacists’ input to improve patient outcomes Protocol for an observational study. BMJ Open.

[20] Tangiisuran B, Scutt G, Stevenson J (2014). Development and validation of a risk model for predicting adverse drug reactions in older people during hospital stay: brighton adverse drug reactions risk (BADRI) model. PLoS ONE.

[21] Akutmodtagelsen – Hillerød. Available from: https://www.nordsjaellandshospital.dk/afdelinger-og-klinikker/akutafdelingen/akutmodtagelse-og-akutklinikker/Sider/akutmodtagelse-hilleroed.aspx. Accessed 24 Feb 2022.

[22] Almarsdóttir AB, Haq R, Nørgaard JDSV (2022). Prioritizing patients for medication review by acute ward pharmacists a mixed methods study in a Danish hospital. Int J Clin Pharm.

[23] Saedder EA, Lisby M, Nielsen LP (2016). Detection of patients at high risk of medication errors: development and validation of an algorithm. Basic Clin Pharmacol Toxicol.

[24] Bonnerup DK, Lisby M, Sædder EA (2020). Effects of stratified medication review in high-risk patients at admission to hospital: a randomised controlled trial. Ther Adv Drug Saf.

[25] Falconer N, Nand S, Liow D (2014). Development of an electronic patient prioritization tool for clinical pharmacist interventions. Am J Health Syst Pharm.

[26] Falconer N, Liow D, Zeng I (2017). Validation of the assessment of risk tool: patient prioritisation technology for clinical pharmacist interventions. Eur J Hosp Pharm.

[27] Law LBK nr 1083 af 15/09/2017 §14 stk.2. Available from: https://www.retsinformation.dk/eli/lta/2017/1083. Accessed 31 Aug 2022.

[28] Landis JR, Koch GG (1977). The measurement of observer agreement for categorical data. Biometrics.

[29] Kornholt J, Christensen MB (2020). Prevalence of polypharmacy in Denmark. Dan Med J.

[30] Page AT, Falster MO, Litchfield M (2019). Polypharmacy among older Australians, 2006–2017: a population-based study. Med J Aust.

[31] Loikas D, Wettermark B, von Euler M (2013). Differences in drug utilisation between men and women: a cross-sectional analysis of all dispensed drugs in Sweden. BMJ Open.

[32] Høj K, Pedersen HS, Lundberg ASB (2021). External validation of the Medication Risk Score in polypharmacy patients in general practice: a tool for prioritizing patients at greatest risk of potential drug-related problems. Basic Clin Pharmacol Toxicol.

[33] Andersen TS, Gemmer MN, Sejberg HRC (2022). Medicines reconciliation in the emergency department: important prescribing discrepancies between the shared medication record and patients’ actual use of medication. J Pharm.

