# Supplementary material for: Comparison of prioritisation algorithms for the selection of patients for medication reviews in the emergency department: a cross-sectional study
Source: Int J Clin Pharm. 2023 Apr 20;45(4):884–92. doi: 10.1007/s11096-023-01582-0 (PMC10366030; doi:10.1007/s11096-023-01582-0)
Supplement: Supplementary file 1 — Supplementary file1 (PDF 121 KB) [file 11096_2023_1582_MOESM1_ESM.pdf]

Comparison of prioritisation algorithms for the selection of patients for medication reviews in the emergency department: a cross-sectional study

International Journal of Clinical Pharmacy

Signe Gejr Korup[1], Anna Birna Almarsdóttir[1*], Line Magnussen[2]

[1] University of Copenhagen, Faculty of Health and Medical Sciences, Department of Pharmacy, Social and Clinical Pharmacy Research Group, Universitetsparken 2, 2100 Copenhagen Ø, DENMARK.

[2] Capital Region Hospital Pharmacy, Nordsjællands Hospital, Dyrehavevej 29, 3400 Hillerød, DENMARK.

*Corresponding author

Email: aba@sund.ku.dk

ORCID http:/orcid.org/0000-0002-5354-2976

The modified version of Assessment of Risk Tool used in the study

Patient Profile	
Item	Score
Age > 75 years	10
No registered general practitioner	5
Patient Encounter	
Item	Score
>4 emergency department admissions in the previous 12 months	8
>2 outpatient visits to different specialities in the previous 6 months	8
Admitted patient under care of haematology or renal service	6
Admitted patient with prior discharge in previous 7 days	4
Admitted patient with prior discharge in previous 30 days	2
Clinical Profile – Patients With Chronic Diseases	
Item	Score
Admitted patient with diabetes	4
Admitted patient with chronic obstructive pulmonary disease	4
Admitted patient with congestive heart failure	4
Admitted patient with cerebrovascular disease	4
High-Risk Medications	
Item	Score
>8 regular medications	10
≥1 antiepileptic medications	2
≥1 anticoagulant medications	2
>3 cardiovascular medications	2
≥1 antidiabetic medications	2
≥1 opioid medications	2
≥1 TDM medications (gentamicin, tobramycin, amikacin, vancomycin, phenytoin, and digoxin)	2
Laboratory Values	
Item	Score
White blood cell count of < 3 x 10^9 /L in past 5 days or neutrophil count of < 1.5 x 10^9 /L in past 5 days	4
Potassium concentration of < 3 or > 5 mmol/L in past 5 days	10
Sodium concentration of < 125 or > 155 mmol/L in past 5 days	10
eGFR < 30 mL/min or serum creatinine > 200 µmol/L in past 5 days	8
An INR of > 3.5 in past 5 days or aPPT of > 100 sec in past 5 days	10
TDM medication concentrations in past 3 days, gentamicin > 1.0 µg/mL, tobramycin > 1.0 mg/mL, amikacin > 1.0 mg/L, vancomycin > 25 mg/L, phenytoin > 80 µmol/L, or digoxin > 2.0 nmol/L	10
A positive Clostridium difficile toxin culture in past 5 days	2

TDM = therapeutic drug monitoring, eGFR = estimated glomerular filtration rate, INR = International Normalised Ratio, aPPT = activated partial thromboplastin time
